# Sleep Disorder in Drug Addiction: Treatment With Transcranial Magnetic Stimulation

**DOI:** 10.3389/fpsyt.2019.00848

**Published:** 2019-11-19

**Authors:** Xiangju Du, Weiqian Xu, Xingxing Li, Dongsheng Zhou, Cuilan Han

**Affiliations:** ^1^Psychiatry Department, Ningbo Kangning Hospital, Ningbo, China; ^2^Center of Sleep Medicine, Taizhou 2nd People’s Hospital, Taizhou, China

**Keywords:** TMS (Transcranial Magnetic Stimulation), sleep disorder, drug, addiction disorders, treatment

## Introduction

Drug addiction subjects sleep less and worse, when compared to healthy control population (1). Importantly, drug addiction are 5–10 times more likely to exhibit sleep disorders than others (2). Drugs of abuse directly modify the sleep quality on both current abusers and abstinent dependents; most drugs prolong the sleep onset latency, reduce the sleep efficiency and total sleep time (3, 4). Sleep disorder is prevalent in drug dependents, ranging from opioid, methamphetamine, cannabis, to alcohol abuse patients (5, 6). Addicts usually have common diseases, including depression, bipolar disorder, schizophrenia, and most of these patients have sleep disorders (7, 8). People with sleep disorders tend to self-treat with alcohol and sedatives, which in turn may promote drug abuse and increase the risk of recurrence (9). Sleep disruption is associated with negative mood status, anxiety and often relate to additional drug intake. Targeting sleep disorder in drug dependents could be important in management of clinical symptom severity and prevention of relapse (10).

## Transcranial Magnetic Stimulation (TMS)

Transcranial magnetic stimulation (TMS) is a way to non-invasively stimulate the brain (11). By positioning a coil over the skull, the electrical current passing through the coil could induce alternating magnetic field, which then transform to alternating current in the cortex and the activation of local neural network. The effects of cortical activation could transmit to connected brain regions, and modulate the release of many different neurotransmitters (12).

Repetitive TMS (rTMS) has been approved for treatment of major depression disorder since 2008, and rTMS has also been employed in management of different psychiatric disorders (13–15). For instance, rTMS could alleviate hallucination in psychotic patients, reduce chronic pain and anxiety, and treat obsessive compulsive disorder (OCD) (16, 17). Recently, rTMS is shown to be effective in addictive disorders as well. High frequency rTMS treatment reduces craving for drugs and intake for cocaine, heroin, methamphetamine, and cannabis (18–23).

On the other hand, rTMS has been used for sleep modulation recently, given the fact that TMS pulse induces slow-wave like activity in the brain (24), and rTMS treatment could re-balance the disrupted network in sleep disorders (25–28).

## rTMS for Sleep Disorder in Addiction

Evidence that rTMS is significantly effective in treating primary insomnia, depression with insomnia and general anxiety with insomnia suggests rTMS intervention is likely to be a promising approach in the treatment of sleep disorder (29–31).

Ensuring the sleep improvement during addiction treatment is important as quality control of detoxification, and prevention for relapse (10, 32). Normalized sleep pattern and circadian rhythm could increase the resilience to addiction (33). Treatment with Benzodiazepines resulted in controversies and with risks for new addiction formation (34). These argues for the need of physical rehabilitation approaches in improving sleep quality for drug dependents.

Emerging studies demonstrated that the potential of rTMS treatment in improving sleep quality and addiction symptoms. One recent study focused on methamphetamine at early abstinence and performed 10 days of high-frequency (10 Hz) rTMS treatment (totally 20,000 pulses) (35). The results showed that rTMS treatment rather than sham treatment reduced withdrawal symptoms, alleviate depression and anxiety state, and improve sleep quality efficiently, with lasting effects in follow-up measurements. Notably, the changes in craving score correlated linearly to sleep improvement. It is possible that shared neurochemistry changes (e.g. increased dopamine concentration in striatum) underlie these behavioral improvements.

Another study recruited male subjects with methamphetamine abuse history, but at extended period of abstinence (e.g. after 3 months) (36). The subjects reported certain levels of depression, anxiety, and sleep disruption. The authors employed chronic rTMS protocol for 6 weeks, with a total of 180,000 pulses for each subject. Following the treatment, the mood status change correlated to the changes in sleep improvement. These results co-suggest for the potential of rTMS in sleep disorder of addicted subjects.

## Opinion

Taken together, sleep disorder as a prevalent symptom in addiction could be treated with rTMS procedures. Few questions remain to be elucidated: (1) Can rTMS procedure be combined with sleep enhancing drugs, such as Benzodiazepines? (2) What is the most important neurochemical component underlying the effects of rTMS on sleep improvement? (3) What is the optimal protocol for rTMS procedure for sleep disorder and addiction treatment? Future studies are required to validate and expand the use of rTMS for sleep disorders in different drug addiction.

## Author Contributions

XD, WX and XL contributed equally to this work. XD, WX drafted the manuscript. XL revised the manuscript. DZ and CH provided funds. All authors reviewed content and approved the final manuscript for publication.

## Funding

The study was supported by the Zhejiang medical and health science and technology program (2018KY182), the Medical Science and Technology Project in Ningbo (2017A10), and Ningbo municipal innovation team of life science and health (2015C110026).

## Conflict of Interest

The authors declare that the research was conducted in the absence of any commercial or financial relationships that could be construed as a potential conflict of interest.
